# The Use of the Initial National Early Warning Score 2 at the Emergency Department as a Predictive Tool of In-Hospital Mortality in Hemodialysis Patients

**DOI:** 10.7759/cureus.39678

**Published:** 2023-05-30

**Authors:** Habibah Sardidi, Dalal Bawazeer, Mohammed Alhafi, Shadan Alomran, Ghali Sayed

**Affiliations:** 1 Emergency Medicine, King Abdulaziz Medical City Riyadh, Riyadh, SAU; 2 Faculty of Medicine, King Saud Bin Abdulaziz University for Health Sciences College of Medicine, Riyadh, SAU

**Keywords:** bacteremia, quick sequential organ failure assessment (qsofa), national early warning score, dialysis, sepsis

## Abstract

Background

The diagnosis of sepsis in the emergency department (ED) is difficult due to the ambiguous nature of its expression and its non-specific symptoms. Multiple scoring tools have been utilized to detect the severity and prognosis of sepsis. This study aimed to evaluate the use of the initial National Early Warning Score 2 (NEWS-2) at the ED as a predictive tool of in-hospital mortality in hemodialysis patients.

Methodology

We performed a retrospective, observational study to review the records of hemodialysis patients admitted to King Abdulaziz Medical City in Riyadh with suspected sepsis from the 1st of January to the 31st of December 2019 using a convenient sampling technique.

Results

The results showed that NEWS-2 had a higher sensitivity in predicting sepsis compared to the Quick Sequential Organ Failure Assessment (qSOFA) (16.28% vs. 11.54%). However, qSOFA had a higher specificity in predicting sepsis compared to the NEWS-2 scoring system (81.16% vs. 74.14%). It was found that the NEWS-2 scoring system was more sensitive in predicting mortality compared to qSOFA (26% vs. 20%). However, qSOFA was more specific in predicting mortality compared to NEWS-2 (88.50% vs. 82.98%).

Conclusions

Our findings demonstrated that the initial NEWS-2 is a subpar screening tool for sepsis and in-hospital mortality in hemodialysis patients. The use of qSOFA at the time of ED presentation was found to have a relatively higher specificity in predicting sepsis and mortality when compared to NEWS-2. To assess the application of the initial NEWS-2 in the ED setting, additional research should be conducted.

## Introduction

Sepsis is defined as an impaired host response to infection leading to life-threatening organ dysfunction [1]. It is a time-sensitive diagnosis with high morbidity and mortality rates in developed and developing countries [2]. It is one of the leading causes of death, with mortality reaching up to 30-40%. Globally, 30 million people are affected by sepsis every year, with 4 to 6 million resulting in death [2]. Hemodialysis patients have been observed to experience significantly higher annual mortality rates due to sepsis compared to the general population, with estimates ranging from 100 to 300 times higher [3]. While epidemiological studies of sepsis in Saudi Arabia are limited, a recent study conducted at Buraidah Central Hospital in Al-Qassim highlighted the prevalence of sepsis among intensive care patients, reporting a mortality rate of 40.3% among those diagnosed with sepsis [4,5]. By focusing on hemodialysis patients, this study seeks to explore the utility of the initial National Early Warning Score 2 (NEWS-2) as a predictive tool for in-hospital mortality, providing valuable insights specific to this vulnerable patient population.

Early recognition of sepsis and antibiotic administration significantly reduce mortality rates [6]. On the other hand, early resuscitation requires noticing that a patient may have a septic shock (SS). The diagnosis of sepsis in the emergency department (ED) is difficult due to the ambiguous nature of its expression and its non-specific symptoms [7]. Multiple scoring tools have been utilized to detect the severity and prognosis of sepsis, such as the System Inflammatory Response Syndrome (SIRS), Quick Sequential Organ Failure Assessment (qSOFA), and National Early Warning Score (NEWS) [8]. Nishiwaki et al. externally validated the use of qSOFA in predicting mortality in hemodialysis patients. Their study concluded qSOFA to be a poor predictive tool of mortality and bacteremia in this population [8].

The NEWS was first introduced in the United Kingdom by the Royal College of Physicians in 2012 and then modified to NEWS-2 in 2017 to become the most widely used tool internationally to predict clinical deterioration in hospitalized patients with sepsis [9]. Its use in the ED has been evaluated by Corfield et al. as a predictive tool of adverse outcomes who revealed an association between a high NEWS score and mortality [6]. Furthermore, Brink et al. compared qSOFA, SIRS, and NEWS in 8,204 patients. In their study, the NEWS was found to be the strongest overall predictor of mortality in patients presenting to the ED with suspected sepsis [10]. To our knowledge, limited studies have assessed the applicability of the NEWS-2 in high-risk populations such as hemodialysis patients.

This study aims to evaluate the use of the initial NEWS-2 in the ED as a predictive tool of in-hospital mortality in hemodialysis patients.

## Materials and methods

Study subjects

We performed a retrospective, observational study to review the records of hemodialysis patients admitted to King Abdulaziz Medical City (KAMC) in Riyadh with suspected sepsis from the 1st of January to the 31st of December 2019 using a convenient sampling technique. The inclusion criteria were adult hemodialysis patients (aged 18 years) who received intravenous antibiotics in the ED or had blood cultures ordered within 24 hours of presentation. Exclusion criteria included pregnancy, patients who suffered cardiopulmonary arrest in the ED, patients with a low frequency of hemodialysis (fewer than one session per week) or a combination dialysis regimen (peritoneal dialysis and hemodialysis), and patients with missing parameters required to calculate the NEWS-2 score.

Outcome measures

The diagnosis of sepsis was made based on positive blood cultures obtained within 24 hours of presentation. The primary outcome was overall hospital mortality. The secondary outcome was assessing the efficacy of the NEWS-2 score in comparison to qSOFA in predicting mortality.

Sample size

To calculate the required sample size, G*Power 3.1.9.7 was used. With related f-tests, linear multiple regression (fixed model, R2 deviation from zero) statistical tests were used to calculate the sample size. Using a medium effect size (0.15), α = 0.05, a power of 0.95 and four predictors, a critical F = 2.444, and numerator df = 4, a sample size of 129 was found to be the minimum required number of participants. To avoid any technical issues and dropout problems, another 20% were added to the study sample. Therefore, the final sample size was 154 participants.

Statistical analysis

Data were analyzed using SPSS version 21.0 (IBM Corp., Armonk, NY, USA). This encompassed the assessment of sensitivity, specificity, positive predictive value (PPV), and negative predictive value (NPV). All statistical tests were conducted at a significance level (alpha = 0.05). For categorical variables, the descriptive statistics were shown as frequency and percentage. For numerical variables, data were shown as mean with standard deviation or median with the first and third quartiles.

Ethical considerations

This study was approved by King Abdullah International Medical Research Center, Riyadh, Saudi Arabia (reference number: RYD-21-419812-167356). No ethical consent was needed because this is a retrospective study. We ensured patients’ confidentiality and anonymity by encoding their data without any identification information into a computer protected by a password known only by the Principal Investigator and Co-investigators.

## Results

The results shown in Table 1 represent the sociodemographic and clinical characteristics of the study sample. The mean age of the patients was 58.14 ± 20.6 years. Male patients constituted 52.6% (n = 91) of the enrolled patients, whereas females constituted 47.4% (n = 82). In addition, the results showed that 97.1% (n = 168) were Saudi patients, whereas 2.9% (n = 5) were non-Saudi patients.

**Table 1:** Sociodemographic characteristics of the patients.

Variable	Mean (standard deviation)	Frequency (%)
Age	58.14 (20.6)	
Gender		
Female		82 (47.4)
Male		91 (52.6)
Nationality		
Saudi		168 (97.1)
Non-Saudi		5 (2.9)
Comorbidities		
Diabetes		112 (64.7)
Hypertension		165 (95.4)
Dyslipidemia		19 (11)
Cerebrovascular disease		24 (13.9)
Ischemic heart disease		22 (12.7)
Asthma		4 (2.3)
Heart failure		15 (8.7)
Deep venous thrombosis		6 (3.5)
Was blood culture sent within 24 hours?		
Yes		171 (98.8)
No		2 (1.2)
Were any antibiotics started in the emergency department?		
Yes		102 (59)
No		71 (41)
Where was the patient admitted?		
Ward		166 (96)
Intensive care unit		7 (4)
Was the blood culture positive?		
Yes		104 (60.1)
No		69 (39.9)
Did the patient expire?		
Yes		60 (34.7)
No		113 (65.3)
Days of patient’s expiry	454.6 (393.6)	

Exploring the patients’ comorbidities revealed that 64.7% (n = 112) had diabetes, 95.4% (n = 165) had hypertension, 11% (n = 19) had dyslipidemia, 13.9% (n = 24) had cerebrovascular diseases, 12.7% (n = 22) had ischemic heart disease, 2.3% (n = 4) had asthma, 8.7% (n = 15) had heart failure, and 3.5% (n = 6) had deep venous thrombosis.

The blood cultures were sent within 24 hours for the majority of the enrolled patients (98.8%, n = 171), whereas for 1.2% (n = 2) of the patients, blood cultures were not sent out within 24 hours. About 59% (n = 102) received antibiotics in the ED, whereas 41% (n = 71) did not. In addition, it was found that 17.3% (n = 30), 16.2% (n = 28), and 14.5% (n = 25) of the patients received ceftriaxone, vancomycin, and piperacillin/tazobactam, respectively.

The majority of the patients enrolled in this study were admitted to a ward (96%, n = 166), whereas 4% (n = 7) were admitted to an intensive care unit (ICU). Among the total recruited patients, about 60.1% (n = 104) had a positive blood culture, whereas 39.9% (n = 69) had a negative blood culture. To ensure that it was not a contaminated sample, the confirmation was performed during admission and labeled as a non-contaminated sample. Moreover, *Staphylococcus aureus* and *Klebsiella pneumonia* were the most common pathogens, as they were found in 27% (n = 28) and 13.5% (n = 60) of the patients, respectively. Finally, the results showed that 34.7% (n = 60) of the patients expired during admission.

**Figure 1:**
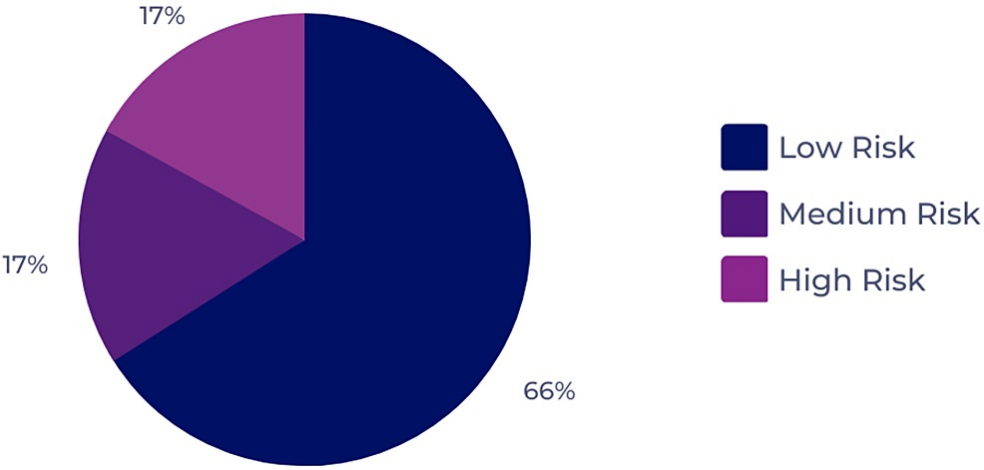
Distribution of the study participants based on the NEWS-2 score and clinical risk. NEWS-2: National Early Warning Score 2

Classifying the patients based on the NEWS-2 score revealed that 66.5% (n = 115) scored 1 to 4 (low risk), whereas 16.8% (n = 29) scored 5-6 (medium risk) and 16.8% (n = 29) scored 7 or more (high risk) (Figure 1).

**Figure 2:**
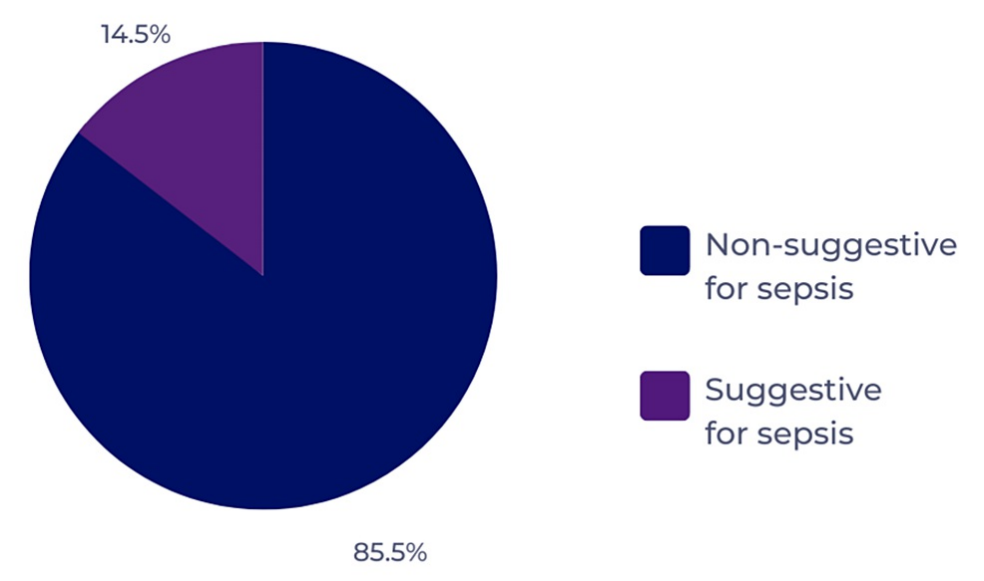
Clinical risk scores for the recruited patients based on qSOFA. qSOFA: Quick Sequential Organ Failure Assessment

Using the qSOFA scoring system revealed that 85.5% (n = 148) of the enrolled patients scored less than 2, which is considered non-suggestive for sepsis, whereas 14.5% (n = 25) scored 2 or more, which is suggestive for sepsis (Figure 2).

The results presented in Table 2 show the sensitivity, specificity, PPV, and NPV for the qSOFA and NEWS-2 scoring systems for sepsis. The results showed that NEWS-2 had a higher sensitivity in predicting sepsis compared to qSOFA (16.28% vs. 11.54%). However, qSOFA had a higher specificity in predicting sepsis compared to the NEWS-2 scoring system (81.16% vs. 74.14%).

**Table 2:** Sensitivity, specificity, PPV, and NPV for different cut-off values for sepsis. NEWS-2: National Early Warning Score 2; qSOFA: Quick Sequential Organ Failure Assessment; PPV: positive predictive value; NPV: negative predictive value; CI: confidence interval

Sepsis	Sensitivity (%) (95% CI)	Specificity (%) (95% CI)	PPV (%) (95% CI)	NPV (%) (95% CI)
NEWS-2	16.28% (9.20%-25.80%)	74.14% (60.96%-84.74%)	48.28% (32.81%-64.08%)	37.39% (33.32%-41.65%)
qSOFA	11.54% (6.11%-19.29%)	81.16% (69.94%-89.57%)	48% (30.9%-63.5%)	37.8% (34.76%-41.02%)
	P ≤ 0.05	P ≤ 0.05	P ≤ 0.05	P ≤ 0.05

The results presented in Table 3 show the sensitivity, specificity, PPV, and NPV for the qSOFA and NEWS-2 scoring systems for mortality. It was found that the NEWS-2 scoring system was more sensitive in predicting mortality compared to qSOFA (26% vs. 20%). However, qSOFA was more specific in predicting mortality compared to NEWS-2 (88.50% vs. 82.98%).

**Table 3:** Sensitivity, specificity, PPV, and NPV for different cut-off values for mortality. NEWS-2: National Early Warning Score 2; qSOFA: Quick Sequential Organ Failure Assessment; PPV: positive predictive value; NPV: negative predictive value; CI: confidence interval

Mortality	Sensitivity (%) (95% CI)	Specificity (%) (95% CI)	PPV (%) (95% CI)	NPV (%) (95% CI)
NEWS-2	26% (14.63%-40.34%)	82.98% (73.84%-89.95%)	44.83% (29.86%-60.80%)	67.83% (63.59%-71.79%)
qSOFA	20% (10.78%-32.33%)	88.5% (81.13%-93.73%)	48.00% (31.01%-65.46%)	67.57% (64.36%-70.62%)
	P ≤ 0.05	P ≤ 0.05	P ≤ 0.05	P ≤ 0.05

## Discussion

This study aims to assess the use of NEWS-2 in the ED setting as a predictive tool for in-hospital mortality and sepsis in hemodialysis patients. The NEWS-2 has several advantages including a low number of variables, quick and easy bedside application, and no requirement for any biological test [11,12]. Our hypothesis was that patients with sepsis would have a higher NEWS-2 score, which would alert ED staff to the possibility of sepsis and prompt them to screen and treat sepsis early.

Our results show that a high NEWS-2 score at the time of ED presentation significantly correlated with patient outcomes, including hospital admission, in-hospital mortality, and ICU admission among hemodialysis patients. To our knowledge, this is the first retrospective study conducted in Saudi Arabia to evaluate the performance of the NEWS-2 as a scoring tool for sepsis in hemodialysis patients presenting to the ED.

The 30-day mortality rate in our study (4.6%) was in line with the 4-6% rate reported in other studies by Brink et al. [10] and Alam et al. [11] which examined similar outcome measures. The prevalence of ICU admissions during hospitalization in our study (4%) was comparable to that reported in a few other studies, ranging from 2.4%-13.1% [10,12-14].

Scoring systems utilized in the ED must have a low threshold to avoid missing sepsis. In our study, both the NEWS-2 and qSOFA reported low sensitivity scores (26% vs. 20%). Brink et al. reported similar results and concluded that NEWS-2 had lower sensitivity resulting in a significant number of false negatives; however, NEWS-2 was found to be a favorable tool for recognizing high-risk and low-risk patients [10]. In our study, qSOFA had a relatively higher specificity in predicting sepsis and mortality when compared with the NEWS-2 (81.16% and 88.5% vs. 74.14% and 82.98%, respectively). This finding is consistent with previous studies that reported qSOFA favors specificity over sensitivity [15-17].

This study had several limitations. First, it was a retrospective, observational study conducted in a single center with a convenient sample, which lacks generalizability. Second, blood cultures and antibiotic initiation were used as a proxy for clinically suspected sepsis. Therefore, we may have included non-septic patients with contaminated blood cultures or bacterial colonization. Third, our study used initially recorded vital signs to calculate the NEWS-2 and q-SOFA scores which may overlook patients with rapid clinical deterioration in the ED. Finally, unknown confounding factors could be present due to the retrospective nature of this study.

## Conclusions

Early recognition of sepsis and antibiotic administration significantly reduce the mortality rate in sepsis patients. In this retrospective, observational, single-center study, we assessed the effectiveness of the NEWS-2 score as a screening tool for sepsis at the time of ED presentation. Our findings demonstrate that the initial NEWS-2 is an insufficient screening tool for sepsis and in-hospital mortality in hemodialysis patients. The use of qSOFA at the time of ED presentation was found to have a relatively higher specificity to predict sepsis and mortality when compared to the NEWS-2. Further studies should be conducted to evaluate the use of the initial NEWS-2 in the ED setting.
